# Delayed Aggressive Local Recurrence of Chondroblastic Osteosarcoma 3 Years After an Excellent Pathological Response: A Case Report

**DOI:** 10.1002/ccr3.71816

**Published:** 2026-01-07

**Authors:** Abdul Hannan, Bilal Aslam, Natasha Hastings, Deepa Lachhman Das, Zahra Sania, Marium Mansoor, Fazeela Bibi, Khalil El Abdi, Samreen Najeeb, Kristen Batten, Manisha Kumari, Said Hamid Sadat

**Affiliations:** ^1^ University College of Medicine and Dentistry University of Lahore Lahore Pakistan; ^2^ School of Medicine St. George's University St. George Grenada; ^3^ Indus Medical College Tando Muhammad Khan Pakistan; ^4^ Women Medical and Dental College Khyber Medical University Peshawar Pakistan; ^5^ Fatima Jinnah Medical University Lahore Pakistan; ^6^ Jinnah Medical and Dental College Kirachi Pakistan; ^7^ Faculty of Medicine and Pharmacy of Rabat Mohammed V University Rabat Morocco; ^8^ St. James School of Medicine Park Ridge Illinois USA; ^9^ Kabul University of Medical Sciences Kabul Afghanistan

**Keywords:** late relapse, local recurrence, oncologic surveillance, osteosarcoma, tumor dormancy

## Abstract

Late recurrence of osteosarcoma after a prolonged disease‐free interval is an uncommon but significant clinical challenge, representing a failure of primary therapy to eradicate microscopic residual disease. We present the case of a 19‐year‐old male diagnosed with chondroblastic osteosarcoma of the proximal tibia who was treated with standard multimodal therapy, including neoadjuvant chemotherapy and limb‐salvage surgery. He achieved wide (R0) surgical margins and an excellent (Huvos Grade IV, > 95% necrosis) pathological response. After a 3‐year disease‐free interval, he presented with an aggressive and extensive local recurrence in the residual tibia, which ultimately necessitated a below‐knee amputation for local control. This case demonstrates that an excellent initial pathological response and wide surgical margins do not preclude the risk of a delayed and highly aggressive relapse in osteosarcoma. It serves as a critical reminder that a “cure” is provisional and underscores the absolute necessity of indefinite, long‐term oncologic surveillance for all survivors. The phenomenon of late recurrence highlights the urgent need for research into tumor dormancy and the development of surveillance tools capable of detecting minimal residual disease.

## Key Clinical Message

An excellent pathological response (Huvos Grade IV) and wide surgical margins in osteosarcoma do not preclude aggressive, delayed local recurrence. This case highlights the clinical reality of tumor dormancy and mandates indefinite, lifelong oncologic surveillance for all survivors, as a “cure” must be considered provisional.

## 1 Introduction

Osteosarcoma is the most common primary malignant bone tumor in adolescents and young adults, typically arising in the metaphysis of long bones such as the proximal tibia [1] The standard of care, consisting of a multimodal approach with neoadjuvant chemotherapy, surgical resection, and adjuvant chemotherapy, has significantly improved prognosis, with 5‐year survival rates for localized disease now approaching 70% [2].

Despite these therapeutic advances, disease recurrence remains a primary cause of treatment failure and a significant clinical challenge [3]. Local recurrence typically occurs within the first 2 years following initial treatment, and relapse after a 3‐year disease‐free interval is less common [4]. This period of deceptive quiescence can create a false sense of security for both patients and clinicians, making late recurrences particularly challenging.

Here, we present the case of a 19‐year‐old male with chondroblastic osteosarcoma of the proximal tibia who, after successful limb‐salvage surgery and chemotherapy, experienced a highly aggressive local recurrence 3 years into remission. The objective of this report is to highlight the unpredictable nature of osteosarcoma and underscore the critical importance of indefinite oncologic surveillance, as a prolonged remission does not preclude the risk of a late and aggressive relapse.

## 2 Case History

A 19‐year‐old male presented to our outpatient department with a 3‐month history of a dull, aching pain in his right proximal tibia. The pain was insidious in onset, progressively worsened at night, and was exacerbated by physical activity. He also reported a noticeable swelling just below his right knee, which had been developing over the preceding 2 months. The patient had no significant past medical history, no known comorbidities, and no family history of malignancy. He was an active recreational basketball player but had recently ceased participation due to the escalating pain and discomfort.

On physical examination, a firm, tender, and warm mass measuring approximately 10 cm × 7 cm was palpated over the anteromedial aspect of the proximal tibia. The mass was immobile, appearing fixed to the underlying bone, and the overlying skin was tense but without erythema. While his knee's range of motion was limited by pain, a comprehensive neurovascular examination of the right lower extremity was normal. No regional lymphadenopathy was detected.

## 3 Investigations and Differential Diagnosis

Initial laboratory findings were unremarkable, with the exception of a mildly elevated serum alkaline phosphatase.

**FIGURE 1:**
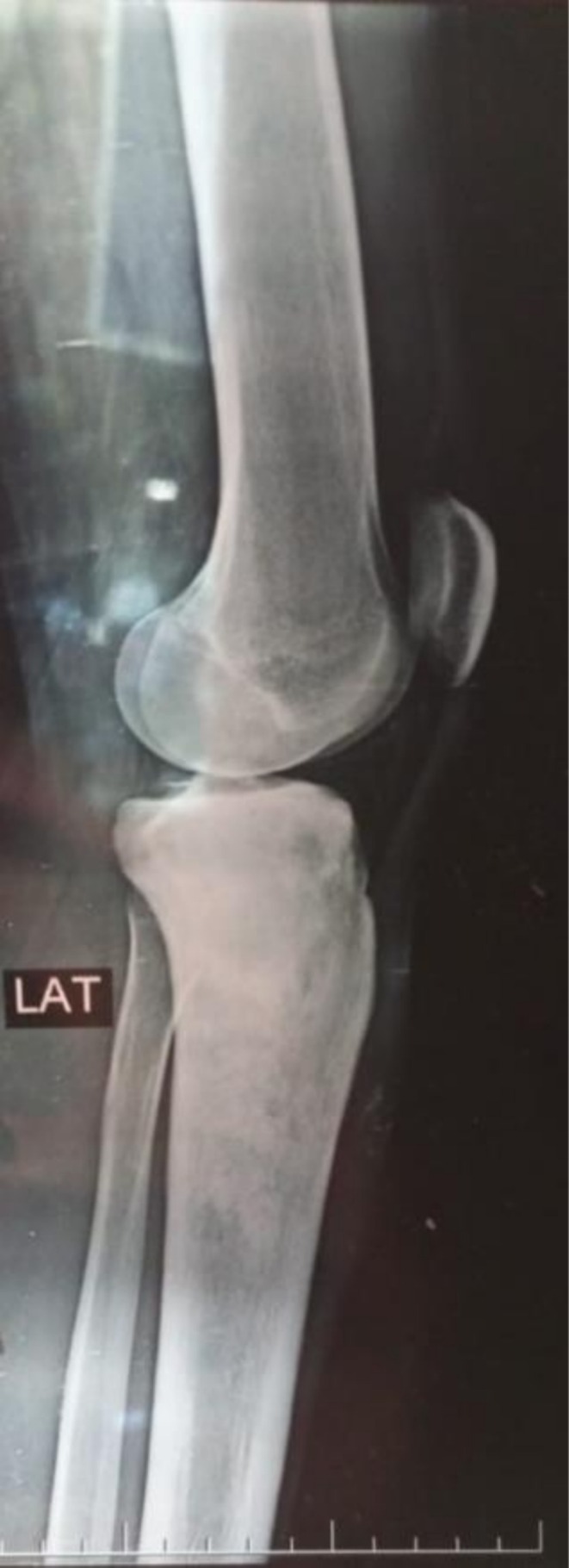
Lateral plain radiograph (X‐ray) of the right knee at presentation. This lateral view reveals a poorly defined, mixed lytic and sclerotic lesion in the proximal tibial metaphysis with typical “sunburst” periosteal reaction, findings highly suggestive of a primary malignant bone tumor. A significant associated soft tissue mass is visible, indicating extensive extra‐osseous tumor extension, a key finding for surgical planning.

**FIGURE 2:**
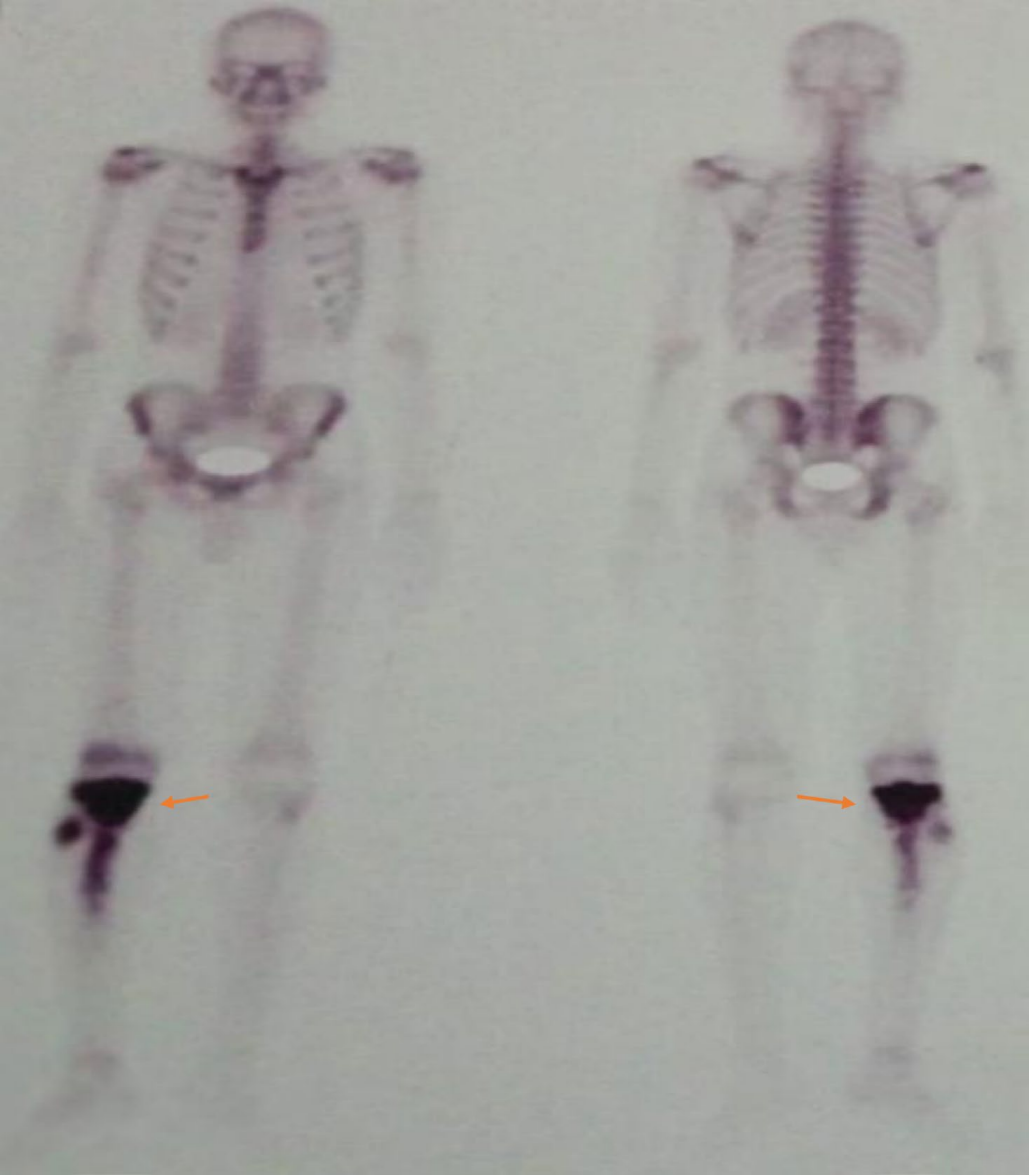
Initial staging with Technetium‐99m bone scintigraphy. Anterior whole‐body bone scan demonstrates intense, heterogeneous radiotracer uptake localized to the metaphyseal‐diaphyseal region of the right proximal tibia (arrow), consistent with the primary chondroblastic osteosarcoma. The scan revealed no evidence of synchronous skeletal metastases at the time of diagnosis.

Plain radiographs of the right leg revealed a metaphyseal osteolytic lesion in the proximal tibia containing areas of sclerosis and a prominent “sunburst” periosteal reaction, findings highly suggestive of a primary malignant bone tumor (Figure 1). For systemic staging, a Technetium‐99m (Tc‐99m) whole‐body bone scan was performed, which demonstrated intense, heterogeneous radiotracer uptake localized to the right proximal tibia, with no evidence of distant skeletal metastases (Figure 2). Magnetic Resonance Imaging (MRI) was conducted to delineate the extent of the disease, revealing a large intramedullary tumor with significant extra‐osseous soft tissue extension; however, the surrounding neurovascular structures appeared uninvolved. A subsequent CT scan provided superior detail of the cortical destruction.

Based on the patient's age and imaging findings, the initial differential diagnosis included osteosarcoma, Ewing sarcoma, and, less likely, an aggressive giant cell tumor or chondroblastoma. To obtain a definitive diagnosis, a CT‐guided core needle biopsy was performed. Histopathological examination revealed malignant spindle‐shaped cells producing a lace‐like osteoid matrix, with distinct focal areas of malignant cartilaginous differentiation. These findings confirmed the diagnosis of Chondroblastic Osteosarcoma.

## 4 Treatment

The patient was treated according to a standard multimodal protocol for high‐grade osteosarcoma. He commenced neoadjuvant chemotherapy with four cycles of high‐dose methotrexate, doxorubicin, and cisplatin (MAP regimen).

**FIGURE 3:**
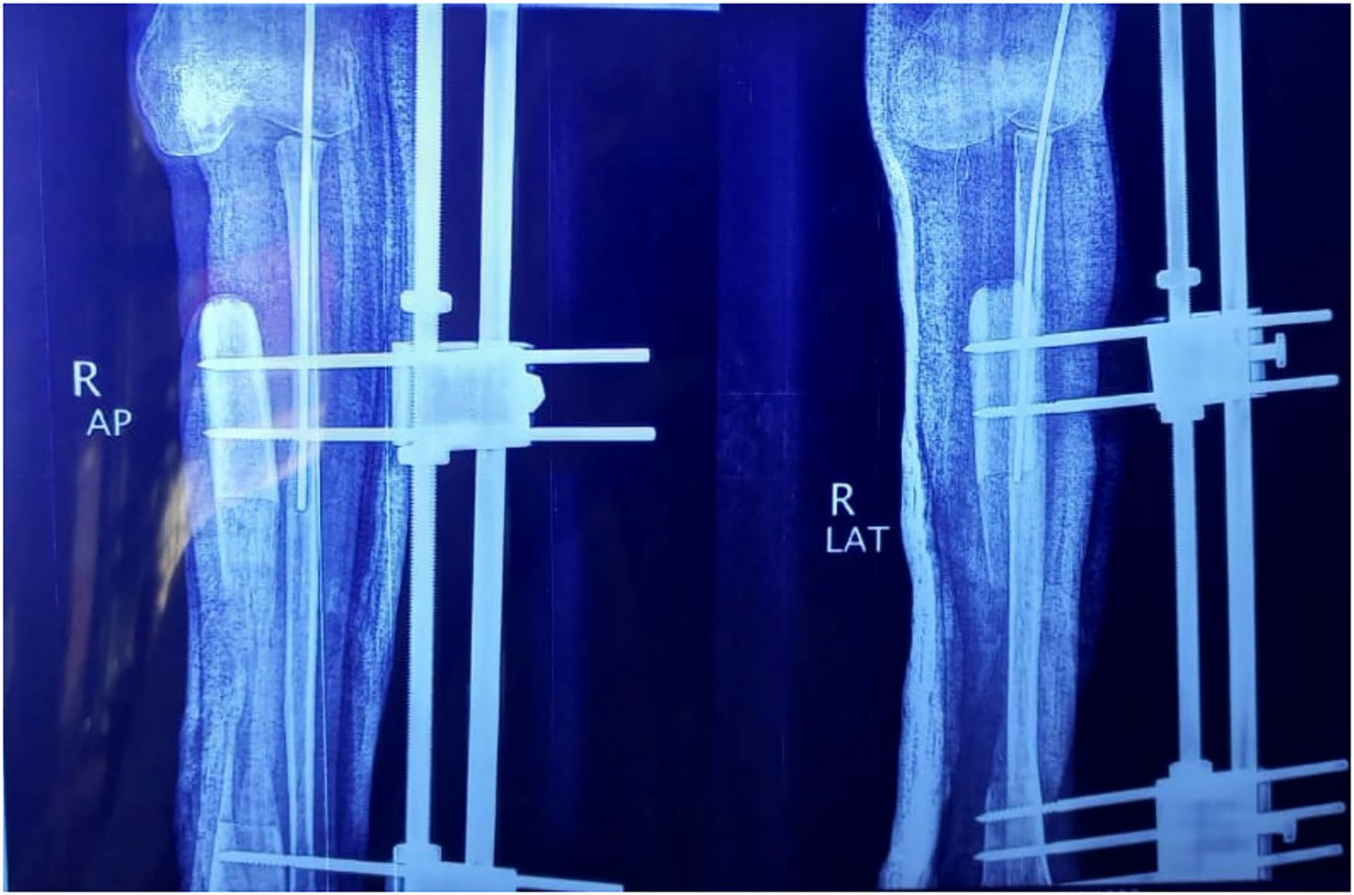
Right leg on plain x‐ray post‐external fixation of distal tibia and fibula and reconstruction with a modular endoprosthesis.

Following the initial chemotherapy, he underwent definitive limb‐salvage surgery. The procedure consisted of a wide en‐bloc resection of the proximal tibia and reconstruction with a modular endoprosthesis (Figure 3). Postoperative histopathological analysis of the resected specimen was crucial; it confirmed wide (R0) surgical margins and showed over 95% tumor necrosis in response to neoadjuvant chemotherapy, corresponding to a Huvos Grade IV (excellent) response. The patient's treatment was completed with two additional cycles of adjuvant MAP chemotherapy.

## 5 Outcomes and Follow‐Up

The patient recovered well from surgery and adjuvant therapy. He was placed on a long‐term surveillance protocol involving regular clinical examinations and imaging. He remained disease‐free for 3 years, with no clinical or radiographic evidence of local recurrence or metastatic disease during this period.

Three years after his initial treatment, he returned to the clinic complaining of new‐onset pain and edema in his distal right leg. Plain radiographs and a subsequent CT scan were performed, which revealed multiple, aggressive lytic lesions with cortical breaks in the shaft of the residual distal tibia, just proximal to the ankle. Given the extent and aggressive nature of this late local recurrence, another limb‐salvage procedure was not feasible. To achieve definitive local disease control, the patient subsequently underwent a below‐knee amputation (BKA).

## 6 Discussion

The clinical course of this patient, defined by a prolonged 3‐year remission followed by an explosive local recurrence, offers a powerful illustration of osteosarcoma's capacity for cellular dormancy and the ultimate failure of our current therapeutic paradigm. This case moves beyond a simple report of late relapse; it serves as a clinical model for the central challenges in modern osteosarcoma management: the persistence of microscopic residual disease, the inherent chemoresistance of tumor subclones, and the urgent need for biologically‐informed surveillance strategies.

A critical analysis of this case must confront the paradox of recurrence despite optimal initial therapy. The patient achieved wide (R0) surgical margins, theoretically ensuring local control, and completed a standard MAP chemotherapy regimen designed to eradicate micrometastatic disease [5]. The relapse, therefore, cannot be attributed to inadequate surgery or omission of care. Instead, it strongly implies the survival of a dormant, chemoresistant subpopulation of cancer cells. The prevailing hypothesis is that initial neoadjuvant chemotherapy acts as a selective pressure, eliminating the bulk of chemosensitive cells while sparing a quiescent, slow‐cycling cellular fraction [6], which may possess stem‐like properties [7]. These cells can persist below the threshold of clinical or radiographic detection, potentially harbored within a protective niche in the tumor microenvironment (TME) [8], only to reemerge years later, often with a more aggressive and therapy‐resistant phenotype [9].

This patient's recurrence also forces a reevaluation of the tumor's immunological landscape, a topic rightly raised by the reviewer. Osteosarcoma is notoriously a “cold” or pauci‐immune malignancy, characterized by low mutational burden [10], infrequent PD‐L1 expression [11], and an immunosuppressive TME dominated by M2‐polarized tumor‐associated macrophages (TAMs) [12]. This context explains the disappointing results of single‐agent immune checkpoint inhibitors in clinical trials [13, 14]. The true challenge is not merely applying immunotherapy, but transforming the TME from an immunosuppressive to an immunopermissive state. Future strategies may involve combining checkpoint inhibitors with agents that deplete TAMs, radiotherapy to induce immunogenic cell death, or cellular therapies like CAR‐T cells engineered to recognize osteosarcoma‐associated antigens. This case exemplifies the patient population for whom such innovative, combination trials are desperately needed, as the relapse demonstrates that conventional cytotoxicity has reached its therapeutic ceiling.

Consequently, our approach to oncologic surveillance requires a fundamental shift from fixed schedules to a more dynamic, risk‐adapted model. The utility of the Technetium‐99m bone scan in detecting this patient's recurrence highlights the value of functional imaging. However, we must look beyond anatomical changes. The future of surveillance lies in detecting minimal residual disease (MRD) through molecular means, such as liquid biopsies that can identify circulating tumor DNA (ctDNA) in the peripheral blood [15]. The presence or reemergence of ctDNA during the surveillance period could serve as a noninvasive biomarker, flagging patients with dormant disease who are at high risk for relapse long before a macroscopic tumor becomes evident. This would allow for preemptive therapeutic interventions and represents the next frontier in secondary prevention for osteosarcoma.

This report is inherently limited by its single‐patient nature. Furthermore, we lack paired genomic sequencing of the primary and recurrent tumors, which could have identified specific mutations or clonal evolution patterns responsible for the aggressive relapse. However, the strength of this case is its stark and unambiguous clinical lesson. It is a clear call to action for the field: we must prioritize research into the mechanisms of tumor dormancy, develop therapies that can eradicate these persistent cells, and innovate surveillance technologies to detect MRD. For the clinician, this case is a humbling reminder that in osteosarcoma, a “cure” is a provisional diagnosis, and lifelong, vigilant surveillance is not just a recommendation—it is an absolute necessity.

## 7 Conclusion

This case of chondroblastic osteosarcoma delivers a critical clinical lesson: a multiyear, disease‐free interval after standard‐of‐care treatment does not guarantee a cure. The aggressive local recurrence observed 3 years after initial remission highlights the deceptive quiescence of this disease and underscores that the risk of relapse extends well beyond the typical 2‐year postoperative window. These findings strongly advocate for indefinite oncologic surveillance and reinforce the need for clinicians to maintain a high index of suspicion for any new symptoms in osteosarcoma survivors, regardless of how long they have been in remission.

## Author Contributions

**Abdul Hannan:** conceptualization, data curation, formal analysis, investigation, writing – original draft. **Bilal Aslam:** conceptualization, formal analysis, writing – original draft. **Natasha Hastings:** conceptualization, data curation, formal analysis, validation, writing – original draft, writing – review and editing. **Deepa Lachhman Das:** formal analysis, writing – original draft. **Zahra Sania:** conceptualization, writing – original draft. **Marium Mansoor:** conceptualization, writing – original draft. **Fazeela Bibi:** conceptualization, formal analysis, writing – original draft. **Khalil El Abdi:** investigation, writing – original draft. **Samreen Najeeb:** conceptualization, writing – original draft. **Kristen Batten:** conceptualization, writing – original draft. **Manisha Kumari:** formal analysis, writing – original draft. **Said Hamid Sadat:** supervision.

## Funding

The authors have nothing to report.

## Consent

Written Informed consent has been obtained from the patient and is available with the other Data upon reasonable request from the corresponding author.

## Data Availability

The data that support the findings of this study are available on request from the corresponding author.
